# Functions of the Alzheimer’s Disease Protease BACE1 at the Synapse in the Central Nervous System

**DOI:** 10.1007/s12031-016-0800-1

**Published:** 2016-07-25

**Authors:** Kathryn M. Munro, Amelia Nash, Martina Pigoni, Stefan F. Lichtenthaler, Jenny M. Gunnersen

**Affiliations:** 1 Department of Anatomy and Neuroscience, School of Biomedical Sciences, The University of Melbourne, Parkville, Melbourne, Australia; 2 Deutsches Zentrum für Neurodegenerative Erkrankungen (DZNE), Munich, Germany; 3 Neuroproteomics, Klinikum rechts der Isar and Institute for Advanced Study, Technische Universität München, 81675 Munich, Germany; 4 Munich Cluster for Systems Neurology (SyNergy), Munich, Germany

**Keywords:** Alzheimer’s disease, BACE1, BACE inhibitors, Synapse, Sez6

## Abstract

Inhibition of the protease β-site amyloid precursor protein-cleaving enzyme 1 (BACE1) is a promising treatment strategy for Alzheimer’s disease, and a number of BACE inhibitors are currently progressing through clinical trials. The strategy aims to decrease production of amyloid-β (Aβ) peptide from the amyloid precursor protein (APP), thus reducing or preventing Aβ toxicity. Over the last decade, it has become clear that BACE1 proteolytically cleaves a number of substrates in addition to APP. These substrates are not known to be involved in the pathogenesis of Alzheimer’s disease but have other roles in the developing and/or mature central nervous system. Consequently, BACE inhibition and knockout in mice results in synaptic and other neuronal dysfunctions and the key substrates responsible for these deficits are still being elucidated. Of the BACE1 substrates that have been validated to date, a number may contribute to the synaptic deficits seen with BACE blockade, including neuregulin 1, close homologue of L1 and seizure-related gene 6. It is important to understand the impact that BACE blockade may have on these substrates and other proteins detected in substrate screens and, if necessary, develop substrate-selective BACE inhibitors.

## BACE1 Is a Therapeutic Target in Alzheimer’s Disease

Alzheimer’s disease is the most common type of dementia and is characterised pathologically by amyloid-β (Aβ) plaque deposition and neurofibrillary tangles. Increased levels of Aβ peptide produced by proteolytic cleavage of the amyloid precursor protein (APP) are associated with the formation of neurotoxic amyloid aggregates and early synapse loss and neuronal dysfunction (Shankar et al. 2008; Selkoe and Hardy 2016). The Aβ peptide is produced by consecutive cleavage of APP by β- and γ-secretases (Lichtenthaler et al. 2011). Additional APP cleavage products, some of which are neurotoxic, are generated by α-, δ- and η-secretases (Lammich et al. 1999; Kuhn et al. 2010; Willem et al. 2015; Zhang et al. 2015). The β-secretase responsible for the proteolytic processing of APP in the brain is β-site APP-cleaving enzyme 1 (BACE1) (Vassar et al. 1999). Processing of APP by BACE1 is the rate-limiting step in the production of Aβ, and therefore, BACE1 is a major therapeutic target for the treatment of Alzheimer’s disease.

BACE1 activity increases in the ageing cortex (Fukumoto et al. 2004), and elevated BACE1 levels can be found in the brains of Alzheimer’s disease patients (Fukumoto et al. 2002; Zhao et al. 2007; Hébert et al. 2008). APP mutations in the human population which increase or decrease APP processing by BACE1 can promote or protect against the development of Alzheimer’s disease, respectively (Mullan et al. 1992; Jonsson et al. 2012). Knockout of BACE1 prevents Aβ production and improves memory in mouse models of Alzheimer’s disease (Cai et al. 2001; Luo et al. 2001; Ohno et al. 2004; Ohno et al. 2006; Ohno et al. 2007). Similarly, the administration of competitive inhibitors of BACE1 improves outcomes in animal models of Alzheimer’s disease and provides the rationale for extending this treatment to patients (Vassar et al. 2014). A number of BACE inhibitors are progressing through clinical trials, the most advanced being MK-8931 from Merck which is currently in phase III trials (Evin et al. 2015; Barão et al. 2016; Mullard 2016). Results from these and subsequent clinical trials will demonstrate how safe and effective BACE inhibitors are for the treatment of Alzheimer’s disease, and may answer questions regarding the optimal level of BACE inhibition and the utility of this approach for treating patients at different stages of disease (e.g. prodromal vs. asymptomatic patients).

However, over the last decade, it has become clear that BACE1 not only cleaves APP but also has a number of other proteolytic substrates which are not known to be involved in the pathogenesis of Alzheimer’s disease but rather have other roles in the central nervous system. Concerns have been raised that mechanism-based side effects of BACE inhibitors may limit their use as therapeutics (Vassar et al. 2014; Evin et al. 2015; Barão et al. 2016). Indeed, recent animal studies have shown that BACE inhibition results in synaptic and cognitive deficits (Filser et al. 2015; Willem et al. 2015) and the production of a newly identified APP peptide, Aη-α, which causes neuronal dysfunction (Willem et al. 2015). Here, we will summarise the synaptic deficits known to result from BACE1 knockout and blockade in mice, and describe key BACE1 substrates that may contribute to this outcome.

## Effects of BACE1 Knockout and BACE1 Inhibition on Synaptic and Neuronal Function

BACE inhibitors are suggested to be most effective when treatment begins very early in the disease pathogenesis (Yan and Vassar 2014), and it is therefore important to know what effect chronic BACE inhibition may have on the brain in the absence of excess levels of Aβ peptide. Constitutive BACE1 knockout (KO) mice provide valuable information about how BACE1 substrates are affected when their proteolytic cleavage is altered. BACE1 KO mice were originally thought to have no significant phenotypes (Luo et al. 2001), but a number of subtle and more severe physiological and behavioural deficits have since been discovered. These include hypomyelination (Hu et al. 2006; Willem et al. 2006), axon guidance errors in the olfactory bulb (Rajapaksha et al. 2011; Cao et al. 2012) and hippocampus (Hitt et al. 2012), seizures (Kobayashi et al. 2008; Hitt et al. 2010), altered astrogenesis and neurogenesis (Hu et al. 2013), increased likelihood of postnatal death and small size (Dominguez et al. 2005), altered insulin sensitivity (Meakin et al. 2012), decreased anxiety (Laird et al. 2005), schizophrenia endophenotypes (Savonenko et al. 2008) and motor deficits (Kobayashi et al. 2008; Cheret et al. 2013). Notably, BACE1 KO mice also exhibit alterations in synaptic number and function. Reduced spine density, including a decreased proportion of mushroom spines, is seen in CA1 pyramidal neurons of the BACE1 KO hippocampus (Savonenko et al. 2008). BACE1 KO mice display altered synaptic plasticity in CA1 and CA3 regions (Laird et al. 2005; Wang et al. 2008; Wang et al. 2014) which is reflected in deficits in hippocampal-dependent spatial reference and working memory (Laird et al. 2005; Kobayashi et al. 2008).

BACE1 KO mice have a complete loss of BACE1, and while the optimum level of inhibition in patients remains to be determined, complete inhibition of BACE1 proteolytic activity is not the aim. Additionally, BACE1 expression is very high during the first postnatal week in mice (Willem et al. 2006) and some phenotypes of BACE1 KO mice are due to altered processing of substrates during development. Therefore, they may be of limited relevance to BACE1 inhibition in the adult brain, even though some neurodevelopmental processes continue in the adult central nervous system or may be reactivated in the diseased brain. Alternatively, it is possible that the lack of BACE1 during development leads to compensatory changes in constitutive BACE1 KOs, and inhibiting BACE1 in the adult brain may have more pronounced effects on particular substrates than predicted from BACE1 KO mice. Fortunately, serious side effects have not been observed in patients in current clinical trials but mechanism-based side effects could potentially counteract the benefits of BACE inhibition.

Recent animal studies indicate that BACE blockade in adulthood can have a negative effect on neuronal function. Firstly, BACE inhibition is associated with a reduction in muscle spindles in adult mice (covered in more detail under the section ‘Neuregulin 1’) (Cheret et al. 2013). Secondly, a study by Willem et al. (2015) demonstrates that BACE inhibitors promote the accumulation of an APP product, Aη-α, which has a detrimental effect on neuronal function. The newly discovered η-secretase was found to cleave the APP protein; this C-terminal fragment (CTF)-η is further processed by BACE1 or α-secretase to produce Aη-α or Aη-β peptides, respectively. Inhibiting BACE in mice leads to increased levels of the Aη-α peptide which is detrimental to neuronal function, altering long-term potentiation (LTP) and reducing neuronal activity (Willem et al. 2015). Therefore, BACE inhibition may simultaneously decrease one neurotoxic APP product (Aβ) while increasing another (Aη-α). Future studies need to clarify whether the increase in Aη-α seen in mice is also observed in humans. Additionally, it will be essential to understand whether and how Aη-α contributes to Alzheimer’s disease. It is currently unclear how the balance of these two APP peptides will ultimately affect the pathological and cognitive changes in Alzheimer’s disease patients. In another recent study, Filser et al. (2015) chronically treated wild-type mice with a BACE inhibitor and identified altered synaptic morphology and function and behavioural deficits. Decreased spine formation on dendrites of cortical pyramidal neurons was observed, which was reversible upon the cessation of treatment. BACE inhibition was shown to decrease neuronal activity and suppress LTP, resulting in memory deficits as assessed by novel object recognition and Y maze spontaneous alternation (Filser et al. 2015). The identification of altered synaptic function and plasticity in the normal adult mouse brain with BACE blockade is significant, as BACE inhibitors may work best if patients are chronically treated from the early stage of the disease. The synaptic and behavioural changes identified by Filser et al. were not attributed to specific BACE1 substrates. There are a number of validated and yet to be validated BACE1 substrates that have known roles in synaptic function and may contribute to the observed results.

Therefore, it is possible that the BACE inhibitors currently being trialled will have some detrimental effects on neuronal function. Inhibition of γ-secretase was previously a viable therapeutic strategy for Alzheimer’s disease, but phase III trials were terminated because of adverse side effects which may have been due to altered processing of the Notch receptor, a γ-secretase substrate (De Strooper 2014). Like BACE1, γ-secretase cleaves a number of substrates in addition to APP. While the BACE inhibition strategy is unlikely to have such severe side effects, there are likely to be mechanism-based side effects associated with the use of BACE inhibitors. Important questions remain: what will the short- and long-term consequences of chronic BACE inhibition be in human patients? Would the effects of BACE blockade on synapses still be reversible in humans if treatment were to be continued for an extended period of time? What is the ideal BACE inhibition strategy which decreases Aβ production while having a minimal impact on other BACE1 substrates? It is important, firstly, to identify the main BACE1 substrates associated with the negative effects on synaptic function and consider whether the BACE inhibitor strategy could be adapted to allow sparing of key substrates, if it should prove necessary.

## BACE1 Substrates Associated with Synaptic Function

Dozens of potential proteolytic substrates of BACE1 have been identified over the last decade, many of which have been identified through proteomic studies (Hemming et al. 2009; Kuhn et al. 2012; Zhou et al. 2012; Hogl et al. 2013; Dislich et al. 2015), and some substrates have been validated in vitro or in vivo by analysing cleaved substrate products in the vertebrate brain or cerebrospinal fluid (CSF). Of these validated substrates, several have known roles in synaptic function and these substrates are discussed below.

### Neuregulin 1

Neuregulin 1 (NRG1) is a member of the neuregulin family of epidermal growth factor (EGF)-like proteins which are ligands for ErbB tyrosine kinase receptors. NRG1 has multiple isoforms that arise from alternative splicing, and these are categorised into six types based on structure (NRG1 types I–VI). All of these isoforms contain an extracellular EGF-like domain; proteolytic cleavage of transmembrane NRG1 is required for the release of the portion of the protein containing this domain, and this soluble form can then bind and activate ErbB receptors (Mei and Nave 2014). NRG1 has a range of biological functions in the central and peripheral nervous systems including the regulation of myelination, radial and tangential neuronal migration (of glutamatergic and GABAergic neurons, respectively) and synaptic plasticity (Mei and Nave 2014). Thus, inhibition or loss of BACE1 activity is predicted to alter NRG1-ErbB signalling and, in fact, a number of the phenotypes seen in BACE1 KO mice are attributed to the lack of BACE1 cleavage of NRG1. For example, BACE1 cleavage of neuronally expressed NRG1 type III is required for normal myelination. BACE1 KO mice, as well as zebrafish lacking BACE1, have peripheral hypomyelination (Hu et al. 2006; Willem et al. 2006; Hu et al. 2008; van Bebber et al. 2013), and central hypomyelination has also been reported (Hu et al. 2006). The proteolytic processing of NRG1 type III and the consequences for myelination have been recently reviewed (Fleck et al. 2012; Hu et al. 2016) and will not be described in detail here.

NRG1-ErbB4 signalling has been intensively studied since the respective genes encoding these proteins were identified as schizophrenia susceptibility genes (Stefansson et al. 2002; Corvin et al. 2004; Mei and Nave 2014; Mostaid et al. 2016). NRG1 heterozygous mice display schizophrenia-like endophenotypes and impaired hippocampal plasticity (Stefansson et al. 2002; O’Tuathaigh et al. 2010), and similar schizophrenia-related endophenotypes are seen in BACE1 KO mice (including impaired pre-pulse inhibition, a greater level of hyperactivity induced by a glutamatergic psychostimulant, cognitive impairments and social recognition deficits; Savonenko et al. 2008). A reduction in PSD-95-associated ErbB4 was also observed in these BACE1 KO mice, supporting the idea that the lack of BACE1 processing of NRG1 and subsequent impairment of NRG1-ErbB4 signalling contributed to the phenotypes observed (Savonenko et al. 2008). Taken together, there is strong evidence from NRG1 overexpression and knockout studies in mice (Mostaid et al. 2016) for the association of altered developmental NRG1-ErbB4 signalling with schizophrenia; however, relatively few studies have addressed the role of BACE1-cleaved NRG1 in the mature brain.

The persistence of NRG1 and ErbB4 expression in the adult central nervous system, including in neurons of the cortex and hippocampus, indicates ongoing roles for NRG1 signalling in maturity (Mei and Xiong 2008). NRG1-ErbB4 signalling is important in regulating synaptic function at both excitatory and inhibitory synapses. For example, ErbB4 is recruited in an activity-dependent manner to the postsynaptic compartment of hippocampal CA1 excitatory synapses where it is activated by soluble NRG1. Binding of NRG1 enhances ErbB4 association with PSD-95 in the postsynaptic scaffold, strengthening glutamatergic synapses through stabilising synaptic AMPA receptors and maintaining dendritic spine synapses (Li et al. 2007). While loss of NRG1-ErbB4 signalling is detrimental (Li et al. 2007; Agarwal et al. 2014), excessive NRG1-ErbB4 activity (as seen in schizophrenia) is also associated with synaptic dysfunction resulting from suppression of LTP-induced NMDA receptor function (Pitcher et al. 2011; Agarwal et al. 2014; Luo et al. 2014).

ErbB4 is also expressed in interneurons in the postnatal and adult brains and is required presynaptically in cortical GABAergic axon terminals for the formation of inhibitory synapses onto pyramidal neurons (Mei and Nave 2014). In dendrites of interneurons, ErbB4 acts postsynaptically at excitatory synapses (Fazzari et al. 2010). Interestingly, deletion of ErbB4 only in fast-spiking interneurons produced neurophysiological and behavioural deficits consistent with schizophrenia endophenotypes (Del Pino et al. 2013). Thus, NRG1 function must be precisely regulated to maintain normal glutamatergic receptor functions at synapses and balanced excitatory-inhibitory neurotransmission in the cortex.

Another important ongoing role of BACE1-processed NRG1 and NRG1-ErbB signalling is the maintenance of muscle spindles, sensory receptors that detect changes in muscle length (Cheret et al. 2013). Chronic treatment of adult wild-type mice with a BACE inhibitor led to a substantial loss of spindles and consequent impairment in motor co-ordination (Cheret et al. 2013). Careful assessment of motor function in patients during chronic BACE inhibitor treatment will be necessary to monitor this potential side effect.

### Sez6 Family

The seizure-related gene 6 (Sez6; also referred to as Seizure 6, Seizure protein 6 and BSRP) family of proteins includes Sez6, Sez6-like (Sez6L) and Sez6-like 2 (Sez6L2). All three family members were identified as BACE1 substrates from a screen that identified proteins shed from cultured neurons using the secretome protein enrichment with click sugars (SPECS) method (Kuhn et al. 2012). Sez6 was validated as a BACE1 substrate, and Sez6 and Sez6L were found to be cleaved predominantly or exclusively by BACE1 (Kuhn et al. 2012). Sez6 is a prime candidate for involvement in the synaptic dysfunction seen with BACE inhibition (Filser et al. 2015) as it is expressed in the adult brain and has known roles in dendrite and spine development (Gunnersen et al. 2007). Given the growing interest in Sez6 family proteins as BACE1 substrates, we have provided a comprehensive coverage of the literature below.

Sez6 messenger RNA (mRNA) is upregulated by neuronal activity (Shimizu-Nishikawa et al. 1995b) and predominantly localised to the central nervous system (Shimizu-Nishikawa et al. 1995b; Herbst and Nicklin 1997). High levels of Sez6 protein are found in the developing and postnatal forebrain (Kim et al. 2002; Gunnersen et al. 2007; Osaki et al. 2011), with Sez6 being detected in the somatodendritic compartment of neurons (Miyazaki et al. 2006; Gunnersen et al. 2007). Sez6 mRNA and protein expression remains relatively high in regions of the adult mouse central nervous system including the cortex, hippocampus, striatum, olfactory tubercule (Herbst and Nicklin 1997; Miyazaki et al. 2006; Gunnersen et al. 2007; Osaki et al. 2011), retina (Gunnersen et al. 2009) and spinal cord (B Graham and J Gunnersen, unpublished data). In constitutive Sez6 KO mice, neurons exhibit morphological alterations including an increased number of dendrites and fewer spines (Gunnersen et al. 2007). Both BACE1 KO and Sez6 KO mice have reduced dendritic spine densities (Savonenko et al. 2008; Gunnersen et al. 2007), deficits in hippocampal-dependent learning (Laird et al. 2005; Gunnersen et al. 2007) and motor deficits (Kobayashi et al. 2008; Gunnersen et al. 2007). The Sez6 gene is highly conserved between mice and humans, and *sez6* mutations and/or altered expression has been associated with febrile seizures (Yu et al. 2007; Mulley et al. 2011), autism spectrum disorder (Cukier et al. 2014; Mariani et al. 2015), intellectual disability (Gilissen et al. 2014) and childhood-onset schizophrenia (Ambalavanan et al. 2016). Elevated, or decreased, levels of Sez6 in the CSF are observed in adult patients with psychiatric disorders (Maccarrone et al. 2013) and Alzheimer’s disease (Khoonsari et al. 2016), respectively.

Sez6 family proteins are each represented by multiple isoforms. Full-length type I transmembrane protein isoforms consist of a signal sequence, a large extracellular or luminal region containing complement subcomponent C1r/C1s, Uegf, Bmp1 (CUB) domains and sushi (also known as short consensus repeat [SCR] or complement control protein [CCP]) domains, a transmembrane region and a short intracellular region containing an NPxY motif, potential phosphorylation sites (Shimizu-Nishikawa et al. 1995b; Miyazaki et al. 2006) and a potential PDZ protein binding domain at the C-terminus (J Gunnersen, unpublished). The presence of CUB and sushi domains suggests that Sez6 family proteins interact with other central nervous system proteins, and an interaction between Sez6 and the protease neurotrypsin (also known as motopsin) has been reported (Mitsui et al. 2013). From the single *sez6* gene, two different transmembrane protein isoforms and a truncated isoform lacking the transmembrane region (secreted) are generated via alternative splicing of the *sez6* mRNA (Shimizu-Nishikawa et al. 1995a; Miyazaki et al. 2006). Sez6 transmembrane and secreted isoforms have opposing actions on dendritic outgrowth in vitro (Gunnersen et al. 2007). Overexpression of the full-length transmembrane Sez6 in neurons lacking endogenous Sez6 inhibits neurite outgrowth while secreted Sez6 promotes it. BACE1 cleaves the transmembrane form of Sez6 to produce a shed ectodomain that is similar to (although larger than) the secreted Sez6 isoform (Kuhn et al. 2012). It will be important to determine whether the BACE1-shed Sez6 ectodomain performs a similar functional role to the secreted isoform, as effective BACE inhibition would be expected to block ectodomain shedding while enhancing the levels of the intact transmembrane form, as shown in mice (Kuhn et al. 2012).

Like Sez6, Sez6L and Sez6L2 are localised to the somatodendritic compartment of neurons (Miyazaki et al. 2006). Sez6L and Sez6L2 mRNA is found throughout the adult mouse brain in regions including the cortex, hippocampus and olfactory bulb (Miyazaki et al. 2006). Mice lacking all Sez6 family members (triple KO mice) have significant motor co-ordination deficits attributed to abnormal climbing fibre innervation in the cerebellum; loss of Sez6L appears to be mostly responsible for this phenotype. The lack of reported phenotypes in the single KO mouse lines in this study indicates a level of functional redundancy between Sez6 family members (Miyazaki et al. 2006). Nevertheless, knockdown of individual Sez6 family members results in decreased calcium spiking frequency and amplitude in cultured neurons (Anderson et al. 2012).

Sez6L was also identified by Kuhn et al. (2012) as a BACE substrate using SPECS. Although not yet validated by other means, further investigation is warranted as, like Sez6, it is predominantly cleaved by BACE1 rather than other proteases (Kuhn et al. 2012). Little is known about its neuronal function although Sez6L mRNA is widely expressed in the adult brain in regions including the cortex and hippocampus (Miyazaki et al. 2006). Sez6L is linked to bipolar disorder (Xu et al. 2013). Outside the central nervous system, *sez6L* genetic variants are associated with multiple cancer types, inflammation and cardiovascular disease. Sez6L2 has been linked to autism (Kumar et al. 2009; Konyukh et al. 2011; Chapman et al. 2015) and is likely to play an important role in central nervous system development. Levels of the shed Sez6L2 ectodomain are decreased in the CSF of BACE1 KO mice (Dislich et al. 2015), although Sez6L2 is likely to be cleaved by other proteases in addition to BACE1 (Kuhn et al. 2012) and cathepsin D is one such protease (Boonen et al. 2016). In this report, Sez6L2 was identified as a transport receptor for cathepsin D, an aspartyl protease found in lysosomes, and was localised to endosomes, the trans-Golgi network and the plasma membrane. Similarly to Sez6, full-length and proteolytically cleaved Sez6L2 exhibited opposing effects on neuronal differentiation and neurite outgrowth in neuroblastoma cells (Boonen et al. 2016). Sez6L and Sez6L2 are expressed in the pancreas where they are BACE2 but not BACE1 substrates, demonstrating the tissue-specific cleavage of these proteins. The CTFs of BACE2-cleaved Sez6L and Sez6L2 are subsequently cleaved by γ-secretase (Stützer et al. 2013), raising the possibility that Sez6 family proteins may undergo this secondary cleavage in the central nervous system.

To understand how Sez6 family proteins contribute to the synaptic deficits seen with BACE inhibition, we need to investigate more fully the functions of uncleaved Sez6 and the BACE1-cleaved Sez6 ectodomain, and learn more about the roles of Sez6L and Sez6L2 in the adult central nervous system.

### CHL1 and L1

L1 (also called L1 cell adhesion molecule) and close homologue of L1 (CHL1) are members of the L1 family of cell adhesion molecules and part of the immunoglobulin superfamily. CHL1 and L1 are type I transmembrane proteins which are widely expressed in neurons, particularly in the developing central nervous system where they facilitate neurite outgrowth, axonal targeting and cell migration (Hillenbrand et al. 1999; Maness and Schachner 2007). CHL1 and L1 cleavage by ADAM proteases alters their function, likely through the release of extracellular fragments or reduction of cell adhesion (Maness and Schachner 2007). CHL1 and L1 were identified as neuronal BACE1 substrates through proteomic screening (Kuhn et al. 2012; Zhou et al. 2012), and they have been validated as physiological BACE1 substrates in vivo (Hitt et al. 2012; Kuhn et al. 2012; Zhou et al. 2012; Dislich et al. 2015).

Neuronal CHL1 is enriched in the axonal membrane (Leshchyns’ka et al. 2006), and co-localisation with BACE1 is observed in growth cones in vitro and pre-synaptic terminals in the postnatal mouse brain (Hitt et al. 2012). Similar hippocampal mossy fibre and olfactory sensory neuron axon guidance defects are observed in constitutive CHL1 KO (Montag-Sallaz et al. 2002) and BACE1 KO mice (Rajapaksha et al. 2011; Cao et al. 2012; Hitt et al. 2012), suggesting that BACE1 processing of CHL1 is required for normal axon targeting. BACE1 cleavage of CHL1 has been shown to contribute to growth cone collapse through interactions of the CHL1-CTF with the axonal guidance molecule semaphorin 3A (Barão et al. 2015).

CHL1 expression decreases following development but persists in the mature central nervous system (Hillenbrand et al. 1999), indicating an ongoing function of CHL1 in the adult brain. CHL1 has a demonstrated role in synaptic transmission: CHL1 accumulates in the pre-synaptic membrane and, through an association of the CHL1 intracellular domain with the chaperone Hsc70, regulates the uncoating of clathrin-coated synaptic vesicles (Leshchyns’ka et al. 2006). Conditional KO mice, in which CHL1 was ablated in excitatory forebrain neurons following postnatal development, exhibited an impaired working memory duration (Kolata et al. 2008) indicating a role for CHL1 in this process in the mature brain. Similarly, L1 is expressed in neurons of the adult brain including the cerebral cortex and hippocampus (Hillenbrand et al. 1999; Horinouchi et al. 2005). Conditional KO mice in which L1 is deleted in mature excitatory forebrain neurons do not display the severe developmental abnormalities seen in constitutive L1 KO mice (Maness and Schachner 2007). However, conditional L1 KO mice display increased basal excitatory activity in hippocampal CA1 and use different search strategies in the Morris water maze, indicating an alteration in place learning (Law et al. 2003).

### APP Family

The APP peptide Aη-α, while not a BACE1 product, is detrimental to neuronal function and is increased in the presence of BACE inhibitors as discussed previously (Willem et al. 2015). Full-length APP and its peptide products also have non-toxic physiological roles related to cell adhesion, intracellular signalling, synaptic function and more, reviewed in detail elsewhere (Müller and Zheng 2012; Nhan et al. 2015). Cleavage of APP by α-secretase or BACE1 leads to the release of soluble ectodomains APPsα and APPsβ, respectively. APPsα has synaptotropic and neuroprotective properties; however, APPsβ has not been shown to have the same potent effect on synapses (Hick et al. 2015; Nhan et al. 2015). The cytoplasmic domain of APP also has a demonstrated role in normal synaptic function (Klevanski et al. 2015). Within the same gene family are amyloid precursor-like protein 1 (APLP1) and amyloid precursor-like protein 2 (APLP2), expressed in the adult brain (Lorent et al. 1995). APLP1 and APLP2 are also type I transmembrane proteins that undergo cleavage by secretases, including BACE1 (Eggert et al. 2004; Li and Südhof 2004; Hogl et al. 2011), to produce a soluble ectodomain, intracellular domain and other (non-Aβ) peptide fragments. Neuronal APLP1 is almost exclusively cleaved by BACE1 (Kuhn et al. 2012); however, relatively little is known about its function. Constitutive APLP1 KO mice display altered synaptic transmission in the dentate gyrus, but to date, there is no clear role for APLP1 in synaptic plasticity (Vnencak et al. 2015). Further study is needed to determine how BACE processing affects APLP1 function.

### Additional BACE1 Substrates

Other potential BACE1 substrates with known roles in synaptic function have been identified in proteolytic screens but await validation in vitro or in vivo. For example, neuroligin 1α, neuroligin 2, neuroligin 4 and neurexin 1α, well-known synaptic cell adhesion molecules, have been identified as likely neuronal BACE1 substrates (Kuhn et al. 2012; Bemben et al. 2015). BACE1 substrates also include proteins that influence the excitability of neurons, including the β-subunits of voltage-gated sodium channels (Wong et al. 2005; Kim et al. 2007; Gersbacher et al. 2010) and subunits of voltage-gated potassium channel proteins, KCNE1 and KCNE2 (Sachse et al. 2013). The BACE1 substrate contactin-2 (Kuhn et al. 2012; Zhou et al. 2012) is a cell adhesion molecule that, together with CASPR2, maintains voltage-gated potassium channels at juxtaparanodal regions of myelinated axons (Poliak et al. 2003). Additionally, while BACE1 primarily acts as a protease, BACE1 can interact non-proteolytically with voltage-dependent sodium channels (Huth et al. 2009) and KCNQ potassium channels (Hessler et al. 2015). Therefore, BACE blockade may alter neuronal excitability through multiple mechanisms.

## Neuronal Localisation of BACE1

Identifying the subcellular compartments in which BACE1 interacts with APP and its additional substrates may promote the development of BACE inhibitors that are ‘substrate sparing’, if adverse mechanism-based side effects of this treatment are found.

BACE1 is expressed in neurons in multiple areas of the healthy and diseased adult brain, including the cortex and hippocampus (Vassar et al. 1999; Fukumoto et al. 2002). In human and mouse neurons, BACE1 is detected in the axonal and somatodendritic compartments; in mouse neurons, BACE1 is transported to, and enriched in, axons (Buggia-Prévot et al. 2013; Buggia-Prévot et al. 2014). BACE1 is prominent in pre-synaptic terminals, and levels are high in the terminals of dystrophic neurites surrounding Aβ plaques (Laird et al. 2005; Zhao et al. 2007; Kandalepas et al. 2013; Sadleir et al. 2016).

BACE1 activity is greatest at acidic pH and can be indirectly inhibited with drugs, such as bepridil, which alkalize the membrane-proximal areas of acidic organelles (Mitterreiter et al. 2010). A recent study by Das et al. (2016) determined that the main subcellular interaction sites of BACE1 and APP in cultured hippocampal neurons depend on the neuronal compartment. In dendrites, including dendritic spines, interaction is most frequent in recycling endosomes with fewer interactions occurring in early endosomal, lysosomal and Golgi vesicles. In axons, the majority of BACE1-APP interactions occur in Golgi-derived vesicles (Das et al. 2016). Validated BACE1 substrates have varied subcellular locations. For example CHL1, L1 and contactin-2 are enriched in axons while Sez6 is localised to the somatodendritic compartment (Gunnersen et al. 2007). Sez6 has been shown to be internalised from the surface of cultured neurons and to be present in transferrin receptor (TfR)-positive early/recycling endosomes (Carrodus et al. 2014); however, further experiments are required to determine whether this compartment is the site of the BACE1-Sez6 interaction.

Identification of the BACE1 substrates that are required for normal synaptic function in the mature brain and elucidation of their subcellular locations will help determine strategies to modify the BACE inhibition approach, if required. For example, experimental approaches to decrease Aβ production while minimising the blockade of key BACE1 substrates include altering the trafficking of BACE1 (Kizuka et al. 2015) and using an endosomally targeted BACE inhibitor (Ben Halima et al. 2016). However, it is possible that the latter strategy will not spare all substrates, as Sez6 family proteins are also abundant in endosomes (Miyazaki et al. 2006; Carrodus et al. 2014).

## Summary

BACE inhibitors for the treatment of Alzheimer’s disease are progressing through clinical trials. If this strategy is found to be effective in preventing or reducing cognitive decline in Alzheimer’s disease patients, considerations for the use of BACE inhibitors include identifying the stage at which the treatment should begin, determining the ideal levels of BACE inhibition at different disease stages and investigating the benefit of combining BACE inhibitors with additional therapeutic strategies. Furthermore, a level of caution surrounds the BACE inhibitor strategy as BACE1 has a number of other substrates in addition to APP. It is imperative that we understand the effects of altered BACE1 cleavage of key substrates and, if warranted, aim to adapt the BACE inhibitor strategy to minimise mechanism-based side effects. Therefore, gaining a better understanding of the fundamental roles of BACE1 substrates in the adult brain and how (and in which subcellular compartments) BACE1 cleavage modulates these functions is of the utmost importance.

## Abbreviations

APLP1: Amyloid precursor-like protein 1
APLP2: Amyloid precursor-like protein 2
APP: Amyloid precursor protein
Aβ: Amyloid β peptide
BACE1: β-Site amyloid precursor protein-cleaving enzyme 1
CHL1: Close homologue of L1
CSF: Cerebrospinal fluid
CTF: C-terminal fragment
CUB: Complement subcomponent C1r/C1s, Uegf, Bmp1
EGF: Epidermal growth factor
KO: Knockout
LTP: Long-term potentiation
NRG1: Neuregulin 1
Sez6: Seizure-related gene 6
Sez6L: Sez6-like protein
Sez6L2: Sez6-like protein 2
SPECS: Secretome protein enrichment with click sugars
